# *In-situ* localization and biochemical analysis of bio-molecules reveals Pb-stress amelioration in *Brassica juncea* L. by co-application of 24-Epibrassinolide and Salicylic Acid

**DOI:** 10.1038/s41598-019-39712-2

**Published:** 2019-03-05

**Authors:** Sukhmeen Kaur Kohli, Shagun Bali, Ruchi Tejpal, Vandana Bhalla, Vinod Verma, Renu Bhardwaj, A. A. Alqarawi, Elsayed Fathi Abd_Allah, Parvaiz Ahmad

**Affiliations:** 1 Department of Botanical and Environmental Sciences, Guru Nanak Dev University, Amritsar, Punjab 143005 India; 2 Department of Chemistry, UGC Sponsored Centre for Advanced Studies-II, Guru Nanak Dev University, Amritsar, Punjab 143005 India; 3 Department of Botany, DAV University, Jalandhar, Punjab 144012 India; 4 Plant Production Department, College of Food and Agricultural Sciences, King Saud University, Riyadh, Saudi Arabia; 5 Botany and Microbiology Department, College of Science, King Saud University, P.O. Box 2460, Riyadh, 11451 Saudi Arabia; 6 Department of Botany, S.P. College, Srinagar, 190001 Jammu and Kashmir India

## Abstract

Lead (Pb) toxicity is a major environmental concern affirming the need of proper mitigation strategies. In the present work, potential of combined treatment of 24-Epibrassinolide (24-EBL) and Salicylic acid (SA) against Pb toxicity to *Brassica juncea* L. seedlings were evaluated. Seedlings pre-imbibed in EBL (0.1 mM) and SA (1 mM) individually and in combination, were sown in Pb supplemented petri-plates (0.25, 0.50 and 0.75 mM). Various microscopic observations and biochemical analysis were made on 10 days old seedlings of *B*. *juncea*. The toxic effects of Pb were evident with enhancement in *in-situ* accumulation of Pb, hydrogen peroxide (H_2_O_2_), malondialdehyde (MDA), nuclear damage, membrane damage, cell death and polyamine. Furthermore, free amino acid were lowered in response to Pb toxicity. The levels of osmoprotectants including total carbohydrate, reducing sugars, trehalose, proline and glycine betaine were elevated in response to Pb treatment. Soaking treatment with combination of 24-EBL and SA led to effective amelioration of toxic effects of Pb. Reduction in Pb accumulation, reactive oxygen content (ROS), cellular damage and GSH levels were noticed in response to treatment with 24-EBL and SA individual and combined levels. The contents of free amino acid, amino acid profiling as well as *in-situ* localization of polyamine (spermidine) was recorded to be enhanced by co-application of 24-EBLand SA. Binary treatment of 24-EBL and SA, further elevated the content of osmoprotectants. The study revealed that co-application of combined treatment of 24-EBL and SA led to dimination of toxic effects of Pb in *B*. *juncea* seedlings.

## Introduction

Contamination of environment with heavy metals is one of the major concerns of the environmentalist in developing and developed countries. Un-controlled addition of heavy metals to the soil has led to far reaching effects on agriculture, as a result of effect on food safety, economic value and uptake by plants and humans^1^. Various metals and metal oxides nanoparticles have been reported to be deleterious for plants^2^. Heavy metal toxicity in plants hampers growth, efficacy of photosynthetic apparatus, senescence and functioning of specific enzymes^3–9^. Lead (Pb) is considered to be one of the most abundant environmental pollutants and enters the environment through anthropogenic addition and consequently cause contamination of biocoenosis and biotopes^10,11^. Once Pb reaches the interior of roots, it gets accumulated in root cells or is translocated to the aerial parts^12–14^. Due to highly toxic nature of Pb, variable symptoms are observed in effected plants including necrosis, chlorosis, growth inhibition, senescence and enhanced generation of reactive oxygen species (ROS) such as hydrogen peroxide (H_2_O_2_), superoxide anion (O_2_^−^), hydroxyl ion (HO), singlet oxygen (O) and nitric oxide (NO) etc^15,16^.

To circumvent metal toxicity, plants have developed discrete strategies by which toxic metal ions are effluxed, retained in roots or are transported to the other parts of plant^3^. These strategies are broadly classified into avoidance and detoxification mechanisms^17^. Plants synthesize certain antioxidative enzymes such as superoxide dismutase (SOD), catalase (CAT), guaiacol peroxidase (POD), ascorbate peroxidase (APOX) and glutathione reductase (GR) and non-enzymatic antioxidants (glutathione, cysteine, ascorbic acid, tocopherol etc.)^18,19^. A few avoidance mechanisms include enhanced accumulation of metal chelating compounds, phenolic compounds and osmoprotectants^20^. More recently, use of exogenous application of plant growth regulators (PGRs) to provide protection to plants against oxidative stress has gained attention^8,9,21,22^. Various phytohormones including auxins (AUX), gibberellins (GBs), ethylene (ET), brassinosteroids (BRs), jasmonic acid (JA) and salicylic acid (SA) have been studied for their positive potential to promote growth and elevate tolerance of plants to heavy metal toxicity^23,24^.

Numerous studies have reported positive potential of BRs as stress protective agents^25,26^. They have been reported to alleviate metal stress in yellow mustard^27^, raddish^28^, cucumber^29^ tomato^30^, Indian mustard^31^ and maize^32^. Similarly, participation of SA in adaptation of plants to wide array of stresses is largely documented^33,34^. Exogenous supplementation with SA to metal stressed plants led to growth promotion and improved photosynthetic efficacy^35,36^, reduced ROS levels^37^ and altered osmolyte levels^38,39^. It has been studied for its anti-stress potential in tobacco^22^, wheat^40^, potato^41^ and rice^42^. In response to various environmental cues BRs interplays with other plant hormones to regulate plethora of attributes of growth and developmental processes in plants^43^. Interplay between BRs and SA has been reported to counteract various stresses^44,45^, including viral infection^46^, fungal infection^47^, salt and temperature stress^45^.

Adequate amount of foregoing studies have been carried out on amelioration of heavy metal induced toxicity by application of BRs and SA individually. In our previous studies we determined the effects of combination of 24-EBL and SA on some physiological and antioxidative characteristics of *B*. *juncea* seedlings grown hydroponically and under field conditions^48–50^. The present study further extended into the few more biochemical parameters including evaluation of oxidative stress, amino acid levels and osmolytes contents and histochemical analysis and is an attempt to better understand the interactive effect of 24-EBL and SA in heavy metal stress amelioration.

## Materials and Methods

### Plant Material

Indian mustard (*Brassica juncea* L., var. RLC-1) seeds were obtained from Punjab Agriculture University, Ludhiana, Punjab, India. The procured seeds were surface sterilized by rinsing for 1 minute in 0.01% mercuric chloride (HgCl_2_), followed by washing with double distilled water. The seeds were then soaked in different solutions of hormones i.e. EBL (0.1 mM), SA (1 mM) and EBL + SA (0.1 mM + 1 mM) for 6 hr. The seeds were sown in autoclaved petri-plates (10 cm, diameter), lined with *Whatmann* no. 1 filter paper. Various Pb concentrations were prepared in Hoagland’s medium (half strength medium was prepared following the method of Cowgill and Milazzo^51^). The petri-plates labelled as control were supplied with only Hoagland’s nutrient medium. The three different Pb (NO_3_)_2_ (lead nitrate) concentration i.e. 0.25 mM, 0.50 mM and 0.75 mM, were selected on the basis of IC_50_ (50% inhibitory concentration). The seedlings were then raised in seed germinator and were provided with 25 ± 0.5 °C temperature, 175 µmol m^−2^ s^−1^ light intensity, 16 hr photoperiod and 80–90% relative humidity. The seedlings were harvested after 10 days of growth. 3 replicates of each treatment were taken for further analysis.

### Pb Localization

The roots of *B*. *juncea* L. seedlings were stained using Pb specific flouroscent probe Leadmium^TM^ Green AM dye (Invitogen, ThermoFisher Scientific). The roots were placed in Na_2_-EDTA (disodium ethylene diamine tetra acetic acid) (20 mM) for 15 minutes and kept at room temperature. The sections were then washed with double distilled water three times for 10 min each. The stock solution of probe was prepared by adding 50 µL dimethyl sulfoxide directly to the vial of the dye, followed by diluting it 1:10 with 0.85% NaCl (sodium chloride)^52^. The roots were then immersed in this diluted stock for 2 hr at 37 °C. It was followed by washing three times with 0.85% NaCl. The roots were then visualized under confocal laser scanning microscope (Nikon AIR). The observations were made at the excitation wavelength of 488 nm and emission wavelength of 590 nm.

### Oxidative Damage

The oxidative damage was evaluated by estimating contents of superoxide anion, H_2_O_2_ and MDA spectrophotopmetrically and by *in-situ* localization of H_2_O_2_, nuclear damage, membrane damage, glutathione (GSH), MDA and cell viability employing confocal laser scanning microscope and visible compound microscope.

#### Superoxide anion content

The level of superoxide anion was determined by following the method of Wu, *et al*.^53^. The seedling extract was prepared in 50 mM potassium phosphate buffer (PPB) with pH 7.8 and supernatant was obtained by centrifugation at 13, 000 × g for 15 minute at 4 °C. To 0.5 ml of the supernatant 0.5 ml PPB, 0.1 ml of 10 mM hydroxylamine hydrochloride was added. The reaction mixture was incubated for 30 minutes at 25 °C, followed by addition of equal volumes of 7 mM 1-napthylamine and 58 mM of 3-aminobenzenesulphonic acid and was incubated for another 20 minutes. The absorbance of the reaction mixture was read at 530 nm. A standard equation was derived to calculate the content of superoxide anion and standard used was sodium nirite (NaNO_2_).

#### H_2_O_2_ estimation

H_2_O_2_ content was estimated by following method of Velikova, *et al*.^54^. The seedling extract was prepared by homogenizing 100 mg fresh plant samples in 1.5 ml of tri-chloroacetic acid (0.1%). The supernatant was obtained by centrifugation of extract at 12,000 × g at 4 °C for 15 minutes. To 0.4 ml of supernatant, 400 µL of PPB (10 mM) and 800 µL of potassium iodide (PI, 1 M) were added. The absorbance of reaction mixture was read at 390 nm and standard curve was prepared using H_2_O_2_ as a standard.

#### MDA content

The level of MDA was determined by the protocol of Heath and Packer^55^. Fresh seedlings were homogenized in tri-chloroacetic acid (0.1%) and were centrifuged for 20 minutes at 4 °C at 13, 000 × g. To the supernatant, 0.5% thiobarbituric acid and tri-chloroacetic acid (20%) were added. The reaction mixture was then kept in water bath at 95 °C for about 95 °C for 30 minutes, followed by cooling the mixture on an ice bath. The absorbance of the reaction mixture was read at 532 nm and 600 nm and content was calculated using extinction coefficient i.e. 155/ mM/ cm.

#### H_2_O_2_ localization

The method given by Ortega-Villasante, *et al*.^56^ was followed for localization of H_2_O_2_ which was done with 2^1^–7^1^- dichloroflourcien diacetate and was incubated for 30 minutes. The roots were then washed three times for 5 minutes with double distilled water and were mounted on a glass slide. The excitation wavelength was 488 nm and emission wavelength was 530 nm respectively.

#### Visualization of membrane damage

For visualization of membrane damage, propidium iodide (PI) a fluorescent adduct was used. A 50 µM solution of PI was prepared according to the method of Gutierrez-Alcala, *et al*.^57^. The root samples were dipped in PI fluorescent probe for 15 minutes, followed by washing with double distilled water and were then mounted on glas slide. The excitation wavelength was 535 nm and emission wavelength was 617 nm respectively.

#### Nuclear damage

The nuclear damage in the root cells was examined by employing a fluorescent dye i.e. 4,6-diamino-2-phenylindole (DAPI) and was prepared following the method of Callard, *et al*.^58^. The dye was prepared by dissolving 0.1 gm of DAPI in 100 ml of phosphate buffer saline (PBS). The roots were mounted on glass slide and visualized at excitation wavelength of 358 nm and emission wavelength of 461 nm respectively.

#### MDA localization

MDA was visualized by employing schiff’s reagent (a visible dye) following the protocol of Wang and Yang^59^. The roots of 10 days old seedlings were excised and dipped in schiff’s reagent for 15 minutes and was followed by washing with 0.5% potassium metabisulphite prepared in 0.05 M HCl. The washed roots were mounted on glass slide and visualized under visible compound microscope.

#### Cell Viability

The method of Romero‐Puertas, *et al*.^60^ was used for analyzing cell viability of root cells. The non-viable cells were localized by 0.25% Evan’s blue dye. The roots were dipped in visible probe and were kept at room temperature for 10 minutes, followed by washing with double distilled water. The prepared slides were visualized under visible compound microscope.

#### Glutathione tagging

Glutathione localization in roots of *B*. *juncea* was done by protocol of Fricker and Meyer^61^. The roots were treated with 25 µM dye i.e. monochlorobimane (MCB) for 20 minutes. The dye solution containing 5 mM of sodium azide prevents accumulation of MCB-GSH conjugate in the vacuoles by eliminating adenosine triphosphate (ATP) from the cells. The excitation and emission wavelengths of the fluorescent adduct was 351–364 nm and 477 nm respectively.

### Quantitative and Qualitative Estimation of Amino Acids

The quantitative estimation of total free amino acids was done by using spectrophotometer, while qualitative profiling of different amino acids was done by employing Amino acid analyzer (Shimadzu, Nexera X_2_).

#### Total free amino acid content

Total free amino acid content was estimated by following the method of Lee and Takahashi^62^. 100 mg of dried plant sample was extracted by dipping the plant material in 5 ml of 80% ethanol (extractant). The samples were then centrifuged for 20 minutes at 4 °C at 2000 × g, followed by addition of 3.8 ml of ninhydrin reagent to 0.2 ml of supernatant. The reaction mixture was boiled for 12 minutes in a water bath. After boiling, the sample were cooled at room temperature followed by reading the absorbance at 570 nm. The betaine hydrochloride was used as standard for preparation of standard curve.

#### Amino acid profiling

The samples for amino acid profiling were prepared by method of Iriti, *et al*.^63^ with minor modifications. 1 gm of fresh seedlings was crushed in 5 ml of 80% methanol. The samples were then centrifuged at 10,000 × g at 4 °C for 20 minutes. To 1 ml of supernatant, 1 ml of sulphosalicylic acid (6%) was added followed by centrifugation at 10,000 × g at 4 °C for 20 minutes. The prepared samples were filtered through 0.22 µm syringe filter. The 1 µL of this sample was injected in the sample vials of amino acid analyzer and were quantified.

### Polyamine (spermidine) localization

#### Synthesis of Probe

The fluorescent probe employed for localization of spermidine was synthesized by following already reported procedure^64^. Reaction mixture comprising of formyl bonic acid, potassium carbonate, Pd (0) in 60 ml of dioxane-H_2_O and tetrabromo 18-crown-6/tetrabromobennzene were kept in N_2_ atmosphere for 24 hrs and was continuously stirred at 80 °C. After 24 hrs, when the reaction was complete, the mixture was removed under pressure, to procure a residue to which water was added. Extraction of the aqueous layer was carried out by using 20 ml chloroform and the process was repeated thrice. A solid residue was obtained from the chloroform fraction by washing the fraction with water followed by drying by using sodium sulphate and finally was distilled under low pressure. Solid residue was then subjected to column chromatography to obtain pure compound by using ethyl acetate as an effluent. The probe was then crystallized by employing ethanol. The synthesized compound was characterized by 1 H NMR, 13 C NMR and Mass spectroscopic studies.

#### Confocal imaging

A 5 mM concentration of probe (molecular weight- 815.2) was prepared in dimethyl sulfoxide (DMSO). Localization of spermidine was done by following the method proposed by Singh *et al*.^65^ with slight modification. The roots of 10 days old seedlings of *B*. *juncea* were excised and incubated in the fluorescent probe for 30 minutes, followed by washing twice with phosphate buffer saline. The washed roots were then mounted on glass slides and visualized under confocal microscope.

### Osmoprotectants

Osmoprotectant viz. total carbohydrate, reducing sugar, trehalose, glycine betaine and proline content were estimated employing double beam UV-Vis spectrophotometer.

#### Total carbohydrate content

Total carbohydrate content was assessed by method of Hodge and Hofreiter^66^. 100 mg of dried plant samples were dipped in 2.5 N HCl and boiled in a water bath for 3 hr. The samples were then cooled and neutralized by addition of sodium carbonates. The sample volume was made upto 100 ml, followed by centrifugation for 20 minutes at 4 °C at 13, 000 × g. To 1 ml of above extract 4 ml of Anthrone reagent and was heated for 8 minutes. The samples were then cooled and observations were made at 630 nm. Glucose was used as standard.

#### Reducing sugar estimation

The protocol proposed by Miller^67^ was used for estimation of reducing sugar levels. 0.1 gm of dried plant material was extracted with 80% ethanol. 3 ml of 3,5-dinitrosalicylic acid (DNSA) was added to 3 ml of plant extract. DNSA reagent was prepared by dissolving 50 mg sodium sulphite, 1 gm DNSA and 200 mg of phenol crystals in 100 ml NaOH (1%) and the reaction mixture was stored at 4 °C. 1 ml of 40% potassium sodium tartarte was added to the reaction mixture. The absorbance of sample was read at 510 nm. Standard glucose concentrations were used for preparation of graph for estimation of reducing sugar levels.

#### Trehalose Content

The method proposed by Trevelyan and Harrison^68^ were used for estimation of trehalose levels. 500 mg of dried plant material was crushed in 80% ethanol, followed by centrifugation at 5000 × g for 10 minutes at 4 °C. 2 ml of tri-chloroacetic acid (0.5 M) and 4 ml of Anthrone reagent was added to 0.1 ml of supernatant. The absorbance of yellow green color was read at 620 nm. Trehalose was used as standard for preparation of graph of absorbance vs. standard trehalose concentration.

#### Glycine Betaine

Estimation of glycine betaine levels was done by following the protocol of Grieve and Grattan^69^. 0.5 gm of dried plant material was extracted with 5 ml of 0.05% of toluene and distilled water mixture. The reaction mixture was incubated in dark for 24 hr and was filtered. 2 N HCl and 0.1 ml of PI were added to 0.5 ml of extract. The samples were incubated for one and half hr in an ice bath. To the above mixture, 2 ml of ice cold water and 10 ml of 1,2-dichloroethane was added. Upper layer was discarded and absorbance of lower layer was read at 365 nm.

#### Proline content

Proline content was assessed by employing method of Bates, *et al*.^70^. 0.5 gm of fresh seedlings were crushed in 3% of sulphosalicylic acid, followed by centrifugation at 13, 000 × g 4 °C for 20 minutes. 2 ml of ninhydrin reagent (1.56 gm of ninhydrin was dissolved in glacial acetic acid and 6 M ortho-phosphoric acid and was warmed and stored at 4 °C), was added and warmed for 1 hr in water bath. To stop the reaction the test tubes were immediately shifted to ice-bath, followed by addition of 4 ml toluene and shook for 30–40 seconds vigorously. The absorbance of toluene layer was read at 520 nm.

### Statistical Analysis

Data obtained was statistically analyzed by self-coded MS Excel software. The data is presented in the form of mean ± standard deviation (SD). The data was also subjected to two-way analysis of variance (ANOVA) and tukey’s test (honestly significant difference, HSD). Multiple Linear Regression (MLR) was employed to analyze the response of independent variables i.e. Pb, 24-EBL and SA. β regression coefficient values implied relative effect of independent variables X_1_-Pb, X_2_- 24-EBL and X_3_- SA. Following equation was used to evaluate the response:

$${\rm{Y}}={\rm{a}}+{{\rm{b}}}_{1}{{\rm{X}}}_{1}+{{\rm{b}}}_{2}{{\rm{X}}}_{2}+{{\rm{b}}}_{3}{{\rm{X}}}_{3}$$

where,Y is parameter analyzed X_1_, X_2_, X_3_ are independent variables i.e. Pb, 24-EBL and SA b_1_, b_2_, b_3_ are partial regression coefficient due independent variables β_1_, β_2_, β_3_ are regression coefficient values.

## Results

### Pb localization

**Figure 1:**
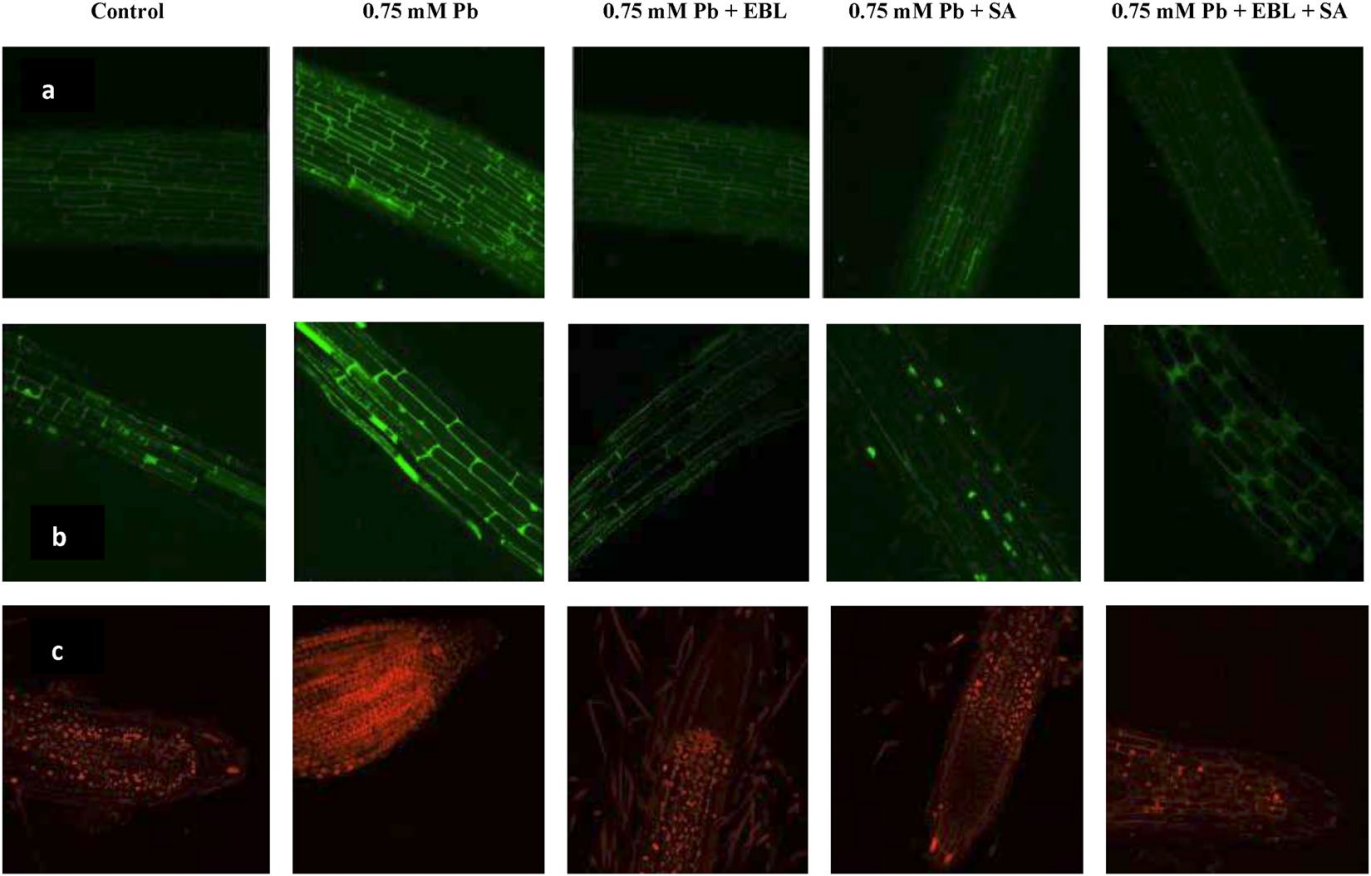
Confocal laser scanning micrographs showing imaging of effect of pre-treatment t with EBL and SA on: (**a**) Pb localization, (**b**) H_2_O_2_ content and (**c**) membrane damage in roots of 10 days old *B*. *juncea* seedlings under Pb stress. Scale bar = 100 μm, (CN = control, Pb = lead, 24-EBL = 24-epibrassinolide, SA = salicylic acid).

The localization of Pb metal ions by Leadmium^TM^ Green AM dye showed green fluorescenece when visualized under confocal microscope. It was observed that the intensity of fluorescence was higher in Pb (0.75 mM) treated seedlings when compared to control. Metal treated seedlings pre-imbibed in 24-EBL and SA, alone and in combination lowered the intensity of green fluorescence. Combined treatment of 24-EBL and SA were found to be most effective (Fig. 1).

### Oxidative damage

#### Superoxide anion content

It was observed that 0.75 mM Pb treatment caused a significant elevation (32.47%) in superoxide anion accumulation in comparison to control seedlings. Pre-treatment of seedling with 24-EBL, SA and combination of 24-EBL and SA resulted in reduction in levels of superoxide anions by 42.33%, 11.79% and 69.36% respectively (Table 1).

**Table 1:** Effect of pre-treatment with combination of 24-EBL and SA on superoxide anion (µg g^−1^ FW), H_2_O_2_ (µM g^−1^ FW) and MDA (mM g^−1^ FW) content in 10 days old *B*. *juncea* seedlings exposed to Pb.

Treatment			Superoxide Anion Content (µg g^−1^ FW)	H_2_O_2_ Content (µM g^−1^ FW)	MDA Content (mM g^−1^ FW)
Pb	24-EBL	SA			
0	0	0	30.80 ± 0.316	2.11 ± 0.107	2.10 ± 0.153
0	0.1 mM	0	21.29 ± 0.499	1.53 ± 0.075	1.02 ± 0.001
0	0	1 mM	25.82 ± 0.255	1.89 ± 0.122	1.54 ± 0.014
0	0.1 mM	1 mM	14.00 ± 0.601	1.36 ± 0.084	0.93 ± 0.020
0.25 mM	0	0	34.01 ± 0.395	2.34 ± 0.098	2.88 ± 0.042
0.25 mM	0.1 mM	0	23.61 ± 0.493	1.70 ± 0.116	1.06 ± 0.013
0.25 mM	0	1 mM	25.96 ± 0.559	1.94 ± 0.142	2.02 ± 0.032
0.25 mM	0.1 mM	1 mM	17.53 ± 0.577	1.34 ± 0.053	0.89 ± 0.023
0.50 mM	0	0	36.03 ± 0.449	4.22 ± 0.096	2.95 ± 0.006
0.50 mM	0.1 mM	0	28.38 ± 0.0393	2.94 ± 0.127	1.49 ± 0.015
0.50 mM	0	1 mM	33.87 ± 0.546	3.32 ± 0.243	1.57 ± 0.015
0.50 mM	0.1 mM	1 mM	18.42 ± 0.638	2.62 ± 0.064	0.58 ± 0.007
0.75 mM	0	0	40.70 ± 0.405	5.80 ± 0.075	3.39 ± 0.476
0.75 mM	0.1 mM	0	23.74 ± 1.006	3.70 ± 0.042	1.60 ± 0.020
0.75 mM	0	1 mM	35.90 ± 0.575	4.21 ± 0.105	2.46 ± 0.046
0.75 mM	0.1 mM	1 mM	12.47 ± 0.638	2.60 ± 0.158	1.07 ± 0.020
F-Ratio_(df 3, 32)_ Treatment			320.26**	1157.52**	205.60**
F-Ratio_(df 3, 32)_ Dose			2917.20**	421.82**	2579.44**
F-Ratio_(df 9, 32)_ Treatment × Dose			99.82**	46.90*	170.46**
HSD			0.299	0.359	0.160
Multiple Linear Regression		β-regression coefficient			Multiple Co-relation Coefficient
		Pb	24-EBL	SA	
Superoxide anion content = 33.62 + 7.829 (Pb) + −1296 (EBL) + −6.824 (SA)		0.27	−0.80	−0.42	0.94***
H_2_O_2_ Content = 2.268 + 3.404 (Pb) + −100.5 (EBL) + −0.633 (SA)		0.79	−0.42	−0.26	0.93***
MDA Content = 2.38 + 0.853 (Pb) + −128.4 (EBL) + −0.679 (SA)		0.29	−0.79	−0.42	0.94***

^*^Data is presented as mean ± SD. Two-way ANOVA, Tukey’s test and MLR analysis was performed * and ** designated significant at P ≤ 0.05 and P ≤ 0.01 respectively. Statistical letters are mentioned HSD.

#### H_2_O_2_ Content

The oxidative burst in plant cells was also assessed by estimating, H_2_O_2_ content. The seedlings raised in 0.75 mM Pb treatment led to increase in H_2_O_2_ levels (174.88%) when compared to control seedlings. Seed soaking treatment of 24-EBL and SA alone resulted in reducing H_2_O_2_ levels by 36.21% and 27.41%, in contrast to 0.75 mM Pb treated seedlings. Co-application of 24-EBL and SA maximally reduced H_2_O_2_ levels by 55.17% respectively when compared to metal (0.75 mM Pb) alone treated seedlings. (Table 1).

#### MDA content

MDA content was significantly elevated in response to Pb treatment. MDA content was enhanced by 38.05% in 0.75 mM Pb treated seedling in comparison to control seedlings. Priming of seedlings with 24-EBL, SA and 24-EBL + SA resulted in reduction in MDA levels by 52.80%, 27.43% and 68.44% respectively in contrast to 0.75 mM Pb treated seedlings (Table 1).

#### H_2_O_2_ imaging

H_2_O_2_ localization was done by employing DCF-DA dye. The green fluorescence was enhanced in Pb (0.75 mM) treated seedlings in contrast to control seedlings, indicating enhancement in H_2_O_2_ levels. 24-EBL and SA individual treatment lowered H_2_O_2_ content as evident by lowered fluorescence. Lowest intensity of green fluorescence was visualized in 24-EBL + SA combination treated seedling implying most effective treatment in reducing H_2_O_2_ (Fig. 1).

#### Membrane damage

PI fluorescent probe was used to observe membrane damage in *B*. *juncea* root cells. PI dye has red fluorescence and the intensity of red color was enhanced in Pb (0.75 mM) treated plants in contrast to control seedlings implying enhanced membrane damage. Pre-treatment of seedlings with 24-EBL, SA and their combination led to reduction in membrane damage as indicated by lowered intensity of red fluorescence (Fig. 1).

#### Nuclear Damage

**Figure 2:**
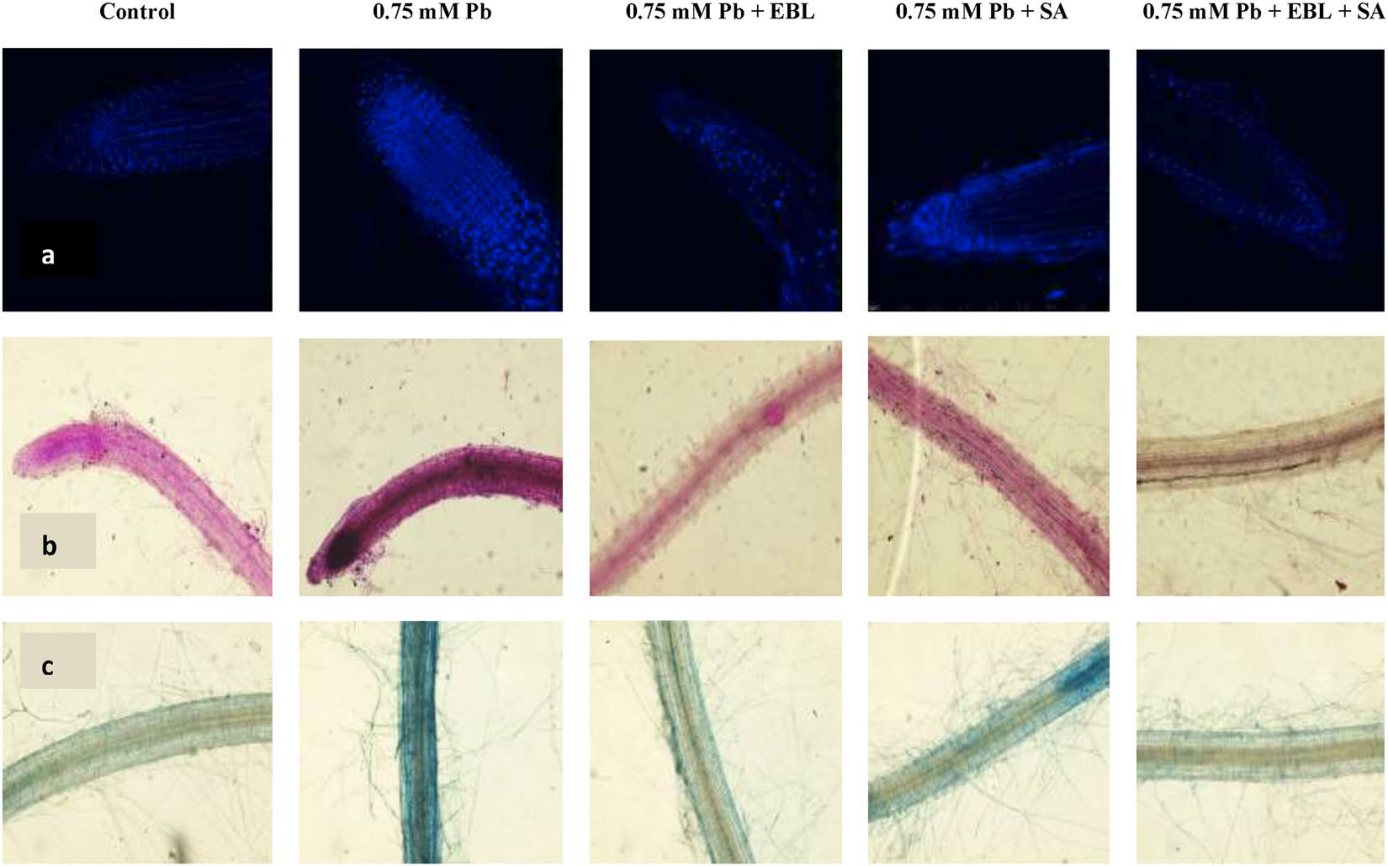
Confocal laser scanning (**a**) and visible (**b**,**c**) micrographs showing imaging of effect of pre-treatment with EBL and SA on: (**a**) nuclear damage, (**b**) MDA content and (**c**) cell viability in roots of 10 days old *B*. *juncea* seedlings under Pb stress. Scale bar = 100 μm (confocal microscope), (CN = control, Pb = lead, 24-EBL = 24-epibrassinolide, SA = salicylic acid).

Nuclear damage was assessed by employing DAPI and has blue fluorescence. The intensity of fluorescence was maximum in Pb (0.75 mM) treated seedlings. 24-EBL and SA alone and in combination showed relative lower intensity of blue color as compared to 0.75 mM Pb treated seedlings. Whereas, 24-EBL + SA combined treatment were the most effective (Fig. 2).

#### MDA visualization

The spectrophotometric results of MDA content were further confirmed by is visualization by schiff’s reagent using visible compound microscope. The Pb (0.75 mM) stressed roots showed higher intensity of pink color of schiff’s reagent in comparison to control seedlings. Priming of seedlings with 24-EBL and SA and their combination showed reduction in MDA levels as compared to metal (0.75 mM Pb) treated seedlings as suggested by lowered intensity of pink color (Fig. 2).

#### Cell Viability

The damaging effect of Pb (0.75 mM) was also assessed by using Evan’s blue dye to visualize cell viability. Lowered viability of cells was observed in (0.75 mM) Pb treated seedlings when compared to control seedlings as implied by darkly stained cells. The viability of 24-EBL, SA and 24-EBL + SA treated seedlings was enhanced as indicated by lowered intensity of Evan’s blue (Fig. 2).

#### Glutathione tagging

**Figure 3:**
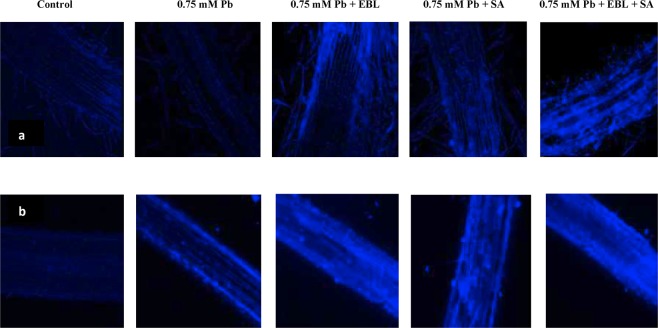
Confocal laser scanning micrographs showing imaging of effect of pre-treatment with EBL and SA on: (**a**) glutathione content and (**b**) polyamine (spermidine) content in roots of 10 days old *B*. *juncea* seedlings under Pb stress. Scale bar = 100 μm, (CN = control, Pb = lead, 24-EBL = 24-epibrassinolide, SA = salicylic acid).

The results of glutathione visualization revealed decline in accumulation of glutathione in 0.75 mM Pb stressed seedlings as evident by reduced fluorescence of MCB dye in comparison to control seedlings. Glutathione levels were enhanced in response to 24-EBL, SA and 24-EBL + SA pre-treatment as indicated by enhanced blue fluorescence (Fig. 3).

### Quantitative and Qualitative amino acids levels

#### Total free amino acid level

**Figure 4:**
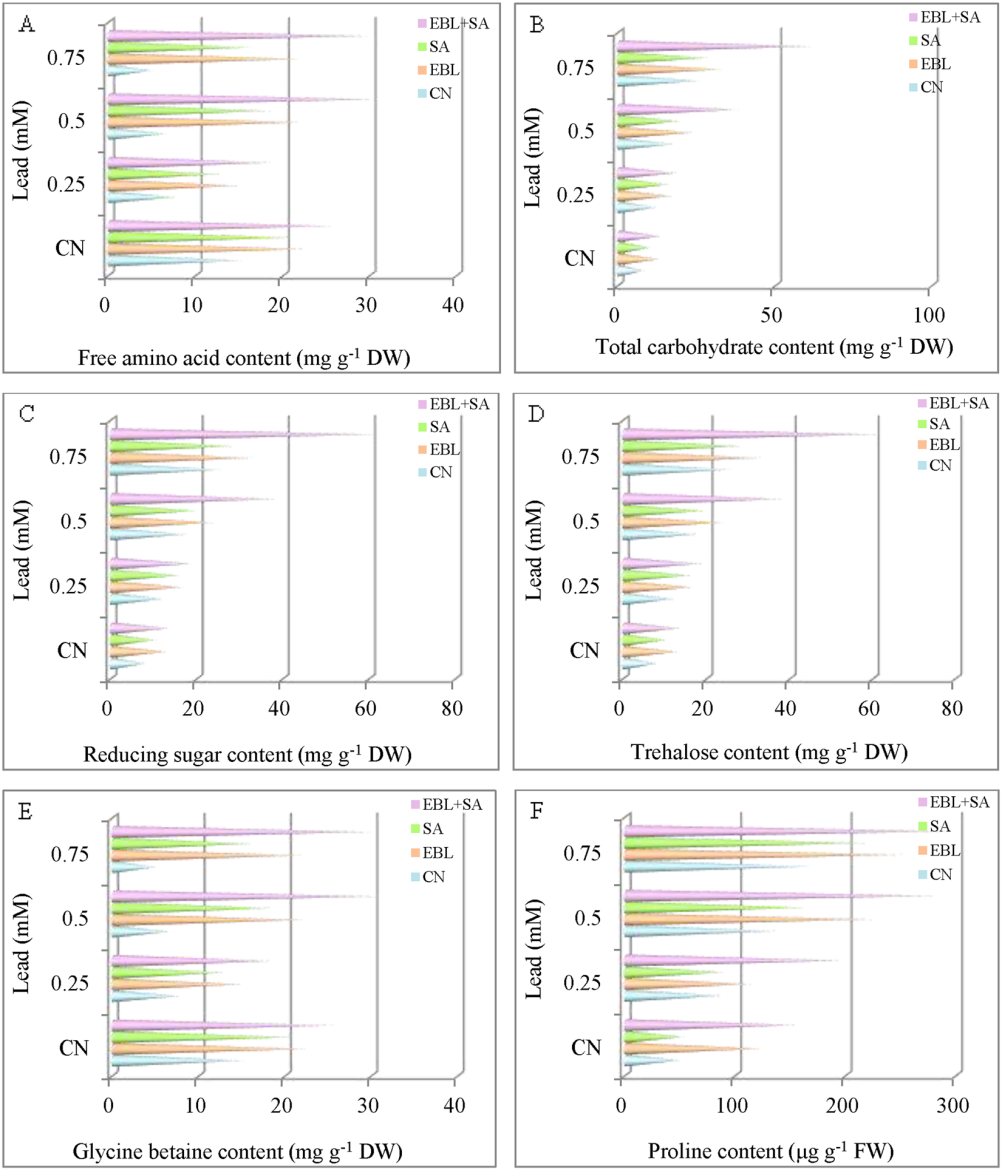
Effect of pre-treatment with EBL and SA on: (**A**) free amino acid, (**B**) total carbohydrate, (**C**) reducing sugar, (**D**) trehalose, (**E**) glycine betaine and (**F**) proline (µg g^−1^ DW) content in roots of 10 days old *B*. *juncea* seedlings under Pb stress, (CN = control, 24-EBL = 24-epibrassinolide, SA = salicylic acid).

The endogenous levels of free amino acids were lowered by 60.49% in 0.75 mM Pb treated seedlings in contrast to un-treated seedlings. Priming of seeds with 24-EBL, SA and their combination led to elevation in free amino acid content by 90.26%, 16.24% and 124.10% respectively, in contrast to 0.75 mM Pb treated seedlings (Fig. 4).

#### Amino acid profiling

A total of 21 amino acids were detected as mentioned in Table 2. Pb metal treatment resulted in decline in levels of specific amino acid including glutamine, GABA, methionine and leucine in comparison to control seedlings. Elevations in content of amino acids were observed when pre-treated with 24-EBL and SA individually as well as in combination. Levels of serine, glutamine, histidine, β-Alanine, GABA, methionine, isoleucine and leucine were further enhanced by co-application of 24-EBL and SA.

**Table 2:** Effect of pre-sowing treatment with combination of EBL and SA on contents of amino acids (µg g^−1^ FW) in 10 days old *B*. *juncea* seedlings under Pb stress.

Treatments									
Pb (mM)	0	0.25 mM	0.25 mM	0.25 mM	0.25 mM	0.75 mM	0.75 mM	0.75 mM	0.75 mM
24-EBL (nM)	0	0	0.1 mM	0	0.1 mM	0	0.1 mM	0	0.1 mM
SA (mM)	0	0	0	1 mM	1 mM	0	0	1 mM	1 mM
**Amino acid content (µg** ^**−1**^ **g of FW)**									
Aspartic acid	—	—	—	—	18.74 ± 4.2	—	13.58 ± 2.8	—	—
Glutamic acid	41.13 ± 5.3	24.48 ± 3.4	55.40 ± 4.2	32.07 ± 3.0	76.45 ± 5.9	—	80.581 ± 4.1	52.44 ± 2.8	46.78 ± 3.3
Serine	46.57 ± 3.4	23.04 ± 1.3	10.11 ± 0.5	14.73 ± 2.9	74.79 ± 2.3	46.86 ± 5.4	32.09 ± 2.9	8.84 ± 0.3	51.68 ± 3.5
Glutamine	150.59 ± 18.2	164.08 ± 6.0	83.16 ± 2.4	117.57 ± 6.1	—	139.48 ± 7.3	136.01 ± 6.0	84.93 ± 3.1	233.31 ± 8.6
Histidine	—	—	—	—	77.52 ± 1.9	38.18 ± 4.7	35.94 ± 4.3	—	45.51 ± 6.2
Glycine	23.29 ± 3.9	26.35 ± 2.7	-	26.68 ± 1.8	23.81 ± 3.3	93.56 ± 5.5	—	—	—
Threonine	90.56 ± 4.6	54.73 ± 5.0	31.63 ± 2.0	41.72 ± 4.3	101.32 ± 3.9	—	73.54 ± 5.2	26.32 ± 4.1	182.07 ± 14.4
Citrulline	14.00 ± 3.1	10.40 ± 1.0	13.97 ± 2.2	14.20 ± 3.2	13.28 ± 1.9	—	—	13.97 ± 0.8	13.23 ± 2.0
Arginine	—	—	—	—	555.97 ± 7.0	—	273.60 ± 6.4	—	281.64 ± 9.2
B-Alanine	38.27 ± 4.1	33.53 ± 2.0	66.41 ± 5.0	43.95 ± 5.9	78.57 ± 7.8	38.27 ± 3.0	42.77 ± 2.3	63.81 ± 2.9	129.58 ± 6.6
GABA	21.82 ± 3.4	10.34 ± 1.1	6.02 ± 0.7	9.76 ± 1.8	—	15.40 ± 0.9	14.53 ± 2.3	—	21.25 ± 3.3
Tryosine	23.75 ± 5.3	10.97 ± 1.6	10.54 ± 1.1	16.03 ± 1.5	54.78 ± 5.7	—	10.86 ± 0.9	8.67 ± 0.6	15.51 ± 4.4
Cystiene	7.14 ± 1.3	22.03 ± 3.1	9.99 ± 0.8	14.81 ± 4.6	12.50 ± 2.2	35.51 ± 4.4	36.79 ± 2.4	8.66 ± 0.2	15.02 ± 0.7
Valine	—	—	—	—	—	25.46 ± 0.8	—	—	—
Methionine	251.70 ± 18.4	336.5 ± 66.3	751.08 ± 14.1	738.07 ± 18.1	484.16 ± 17.2	218.20 ± 7.1	887.53 ± 10.3	714.29 ± 11.3	1156.78 ± 23.5
Phenylalanine	14.70 ± 3.0	12.53 ± 0.8	—	—	—	—	—	—	—
Isoleucine	45.97 ± 4.6	47.18 ± 2.2	78.14 ± 5.2	66.73 ± 4.7	88.78 ± 4.2	67.07 ± 2.1	44.52 ± 6.2	63.23 ± 2.0	94.09 ± 6.3
Leucine	34.26 ± 4.845	43.91 ± 3.1	44.19 ± 3.5	49.00 ± 4.1	55.95 ± 5.4	34.05 ± 3.1	25.31 ± 2.5	44.20 ± 3.0	60.58 ± 8.3
Ornithine	—	—	—	—	8.14 ± 1.1	9.21 ± 0.4	—	—	7.15 ± 0.7
Lysine	10.14 ± 1.421	11.68 ± 0.3	—	10.56 ± 1.3	20.85 ± 2.6	14.84 ± 3.0	10.58 ± 1.6	—	12.95 ± 1.4
Proline	14.19 ± 1.380	136.3 ± 14.8	241.58 ± 20.1	7.72 ± 0.6	25.09 ± 4.4	116.55 ± 10.1	137.48 ± 3.7	227.78 ± 10.2	20.62 ± 1.7

### Polyamine (Spermidine) imaging

*In-situ* localization of spermidine in roots of *B*. *juncea* was done using chemical probe, which showed blue fluorescence. It was observed that, 0.75 mM Pb treated plants had higher levels of polyamine when compared to control seedlings as indicated by darkly stained cells. Individual as well as combined application of 24-EBL and SA led to further elevation in accumulation of polyamines as evidenced by darkly stained cells (Fig. 3).

### Osmoprotectants

#### Total Carbohydrates Content

Pb (0.75 mM) treatment led to significant enhancement (195.60%) in total carbohydrate content in comparison to control seedlings. Seedlings treated with 24-EBL and SA individually and in combination led to further enhancement in levels of total carbohydrates by 179.9%, 17.48% and 207.80% respectively. Combined treatment of 24-EBL and SA was most effective in enhancing total carbohydrate levels (Fig. 4).

#### Reducing sugar content

The seedlings raised under 0.75 mM Pb treatment had elevated levels of reducing sugars by 51.25% in comparison to control. Seedlings primed with in 24-EBL and SA individual treatment led to increase in reducing sugar levels by 222.40% and 146.20% respectively in contrast to 0.75 mM Pb treatment. Co-application of 24-EBL and SA maximally enhanced reducing sugar levels by 246.25% in comparison to metal (0.75 mM) Pb treated seedlings (Fig. 4).

#### Trehalose content

Trehalose levels were significantly elevated in response to Pb treatment by 219.42% in contrast to control seedlings. Priming with 24-EBL, SA and 24-EBL + SA resulted in enhancement in trehalose levels by 28.72%, 8.06% and 140.80% respectively in comparison to 0.75 mM Pb treated seedlings (Fig. 4).

#### Glycine betaine content

Content of glycine betaine was enhanced by 64.15% in Pb (0.75 mM) treated seedlings in contrast to control seedlings. Priming of seeds with 24-EBL and SA individual treatment further enhanced the levels of glycine betaine by 21.07% and 10.92% respectively in comparison to 0.75 mM Pb treated seedlings. Pb (0.75 mM) treated seedlings pre-imbibed in combined treatment of 24-EBL and SA led to most significant elevation i.e. by 40.61% respectively in glycine betaine content (Fig. 4).

#### Proline level

Significant elevation in proline content was recorded in 0.75 mM Pb treated seedlings (237.91%) in comparison to control seedlings. Further increase in proline levels were recorded in 24-EBL, SA and 24-EBL + SA primed seedlings by 50.60%, 31.51% and 76.31% respectively when compared to 0.75 mM Pb treated seedlings (Fig. 4).

## Discussion

Pb is a non-essential element that prominently perturbs plants physiology. It is a less available and low solubility metal which occurs in phosphates, nitrates and sulphate forms^71^. It is a extremely toxic metal and exerts certain critical effects on the physiological and biochemical attributes in plants^72^. *In-situ* localization of Pb ions in roots revealed enhancement in Pb content in response to increment in metal concentration. Similar elevation in Pb content was reported in *Oryza sativa*^73^, *Nicotiana tabaccum*^74^, *Medicago sativa*^75^ and *Triticum aestivum*^76^. The bulk of Pb in soil is absorbed by roots of plants and bind to the carboxyl group of mucilage uronic acids^77^. A very small fraction of Pb is available for plants to accumulate due to the fact that it forms strong complexes with colloidal particles or organic matter^78^. The endodermis of the root cells act as a barrier for movement of Pb from roots to shoots. This may be a possible reason for higher accumulation of Pb in roots when compared to shoots^79^. Plant growth regulators (PGRs) are well studied for their anti-metal stress activities^80^. In present study pre-treatment of seedling with EBL, SA and their combination showed reduced Pb metal localization in root cells. This is attributed to BR stimulated activation of antioxidative defense system, lowered ROS content and improvement in growth of plants^21,27^. Several reports suggest BR-induced reduction in metal upake such as Al in *Phaseolus aureus*^81^, Ni in *Brassica juncea*^31^, Cr in *Raphanus sativus*^82^ and Cu in *Brassica juncea*^83^. Similarly, SA application to *B*. *juncea* seedlings resulted in lowering of Pb ion deposition in root cells. Transportation and accumulation of several metals have been reported to be affected by exogenous application of SA. Decline in metal content i.e. Cd in *Zea mays*^84^, Pb in *B*. *juncea*^85^, As in *Raphanus sativus*^86^ and Ni in *Nicotiana tabaccum*^22^ was reported in SA pre-treated plants. Reduced uptake of Pb in *Chlorella vulgaris* cells has been reported under 24-EBL treatment by Bajguz^87^. He suggested that Pb metal in combination with EBL results in stimulation of phytochelatin de-novo synthesis. Similarly, Kaur *et al*.^88^ suggested that SA treatment leads to reduced metal uptake, possibly due to its ability to reduce oxidative stress and increased membrane stability^88^.

Exposure of *B*. *juncea* plants to Pb in the present study resulted in enhanced synthesis of ROS which further caused cellular damage in plants. Pb treatment resulted in elevation in levels of superoxide anion, H_2_O_2_ and MDA levels. ROS production in response to Pb stress is well documented^89,90^. H_2_O_2_ is easily converted to hydroxyl ion (free radical) by fenton reaction and is membrane permeable and diffusible^91^. The enhanced production of ROS might also enhance the tolerance mechanism in plants^92^. Asada–Halliwell pathway, with the co-ordination of enzymatic and non-enzymatic antioxidants, also plays an instrumental role in the removal of H_2_O_2_. Histological studies carried out also supported the biochemical responses and affirmed enhancement in total ROS, H_2_O_2_ and MDA levels. Furthermore, cellular damage and loss of viability of cells caused by oxidative burst was also observed. The results of present study corroborated with the previous studies conducted on *Ocimum tenuiflorum*^93^, *Vigna radiata*^94^ and *Zea mays*^95^. BRs application resulted in reduction in ROS levels and hence aided in lowering the toxic effects of Pb. Similar findings of lowered ROS production were reported by Sharma and Bhardwaj^83^ in *Zea mays* plants by Choudhary, *et al*.^96^ in *Raphanus sativus*, by Yadav, *et al*.^27^ and by Kohli, *et al*.^49^ in *B*. *juncea*. Reduction in Pb induced MDA, H_2_O_2_ and superoxide anion content by BRs pre-treatment is attributed to their ability to enhance activity of antioxidative defense system and hence scavenging of free radicals^97^. Interplay between SA signaling networks and other signaling cascade has been studied to regulate ROS stimulated cell death^98^. The observation was also affirmed with histological studies which showed reduction in ROS content, nuclear and membrane damage and altered cell viability.

Sulfur is one of the most imperative elements that is incorporated in several biomolecules including amino acids and antioxidants^99^. GSH is one of the sulfur containing antioxidant. Present work further revealed reduction in levels of glutathione in response to Pb toxicity. Our findings corroborated the studies of Okamoto, *et al*.^100^ who reported lowered GSH in *Gongauloux polyedea* exposed to Pb stress. The decline in GSH levels is due to lowered de-novo production of GSH as well as over-utilization of GSH in the synthesis of phytochelatins, pre-requisite for chelation of toxic metal ions^101^. This antioxidant fortifies protein oxidation and acts as a substrate for production of glutathione peroxidase and glutathione-S-transferase^102^. In addition, heavy metals can alter the active sites of the enzymes, thus rendering them nonfunctional^103^. Exogenous application of combination of 24-EBL and SA resulted in enhancement in levels of GSH in the present study. Exogenous application of BRs to stressed plants elevated the contents of glutathione in *Lycopersicon esculentum* exposed to Cd and Pb^30^, *Cicer arietinum* exposed to Cd^104^ and *Vigna radiata* exposed to Al^105^. The possible reasons for BR induced increase in the activities of antioxidative enzymes might be due to their ability to regulate the transcription and/or translation of genes that further mediate the activation, or de novo synthesis of antioxidative enzymes^87^. Similarly, application of SA was also reported to enhance endogenous levels of GSH in *Medicago sativa* plants under Mg stress. SA primed plants had enhanced tolerance to Cd in *Triticum aestivum* plants which was largely co-related to enhanced GSH synthesis. Furthermore, several studies suggested regulation of various enzymes of AsA-GSH (ascorbate-glutathione) cycle by SA application^106,107^. Another possible reason for enhanced activity of antioxidative defense system might be due to BR Signaling Kinase 1 (BSK 1) which is a positive regulator of SA accumulation in stressed plants and eventually results in amelioration of oxidative damage^108^.

Amino acids specifically S containing amino acids have anti-stress and antioxidant properties. These organic amino acids include glutathione and methionine^109^. In the present study, the levels of free amino acids and other amino acids was lowered in response to Pb stress. Amino acids have metal chelating, antioxidant and signaling ability under metal stress^110^. The probable reason for lowered amino acid levels might be due to enhanced chelation of bivalent metal ions with free amino acid which consequently lowers available amino acids^111^. Co-application of BRs and SA resulted in elevation in free amino acid levels. Our findings corroborated with the study carried out by Li, *et al*.^112^ who reported enhancement in amino acids content in response to 24-EBL application. Similarly, application of SA resulted in elevation in levels of amino acids specifically proline, glycine betaine and glutathione in the present study. Few specific amino acids such as proline and glycine betatine also act as osmoprotectants^113^. Combined application of 24-EBL and SA led to enhancement in osmolyte levels in the present study. Similar elevation in glycine betaine levels were observed in response to CO, Fe and Zn stress^114^. Glycine betaine levels are known to alter the membrane permeability of stressed plant cells^115^. Enhanced levels of proline and glycine betaine resulted in further elevation in antioxidative capacities of berries^116^. Proline content significantly elevated by BRs application is well documented^29,82,96,117^. SA also resulted in further enhancement in proline and glycine betaine levels and they aid in detoxification of elevated ROS content, maintenance of membrane stability and enzyme activities^118^. It was observed by Parmar *et al*.^119^, that the main reason for increase in levels of proline content is associated with increase in synthesis of new amino acids under heavy metal stress. Elevation in concentration of osmolytes is considered as an important marker indicating metal stress and have important role in stress mitigation.

*In-situ* localization of polyamines (spermidine) revealed elevation in polyamines accumulation in Pb stressed seedlings. Polyamines specifically spermine and spermidine have antioxidant properties and protect DNA against oxidative damage^120^. Similar to our findings, Groppa, *et al*.^121^ reported elevation in polyamines content in response to Cu and Cd toxicity. Another report suggests similar decline in polyamine accumulation in *Lacuta sativa* plants under Zn stress^122^. Co-application of 24-EBL and SA significantly elevated *in-situ* deposition of polyamines in root cells of *B*. *juncea* in the present study. In our earlier study, Choudhary, *et al*.^123^ reported positive crosstalk between BRs and polyamines in regulating Cu stress in *Raphanus sativus* plants. It was further suggested that synergistic interplay between BRs and polyamines (spermidine) led to enhancement in levels of other polyamines such as putrecine and spermine. Similarly, exogenous application of SA resulted in higher level of polyamines in *Zea mays*^124^ and *Lycopersicon esculentum*^125^.

Sugars and sugar polyols accumulate in response to stress and they act as an imperative biomarker and osmoregulator^126^. Levels of total carbohydrate, reducing sugars and trehalose were significantly increased in response to Pb treatment. Sugars play a vital role in modulating plants osmotic balance and membrane stability in stressed plants^127^. It has been hypothesized that heavy metal toxicity might hinder the metabolic pathway of carbohydrates or it might play a role in the accumulation of photoassimilates because of reduced loading of veins^128^. Plethora of reports suggest enhancement in sugar levels due to Pb toxicity in *B*. *juncea*^48^ and *Raphanus sativus*^129^. It was further suggested by Bhushan and Gupta^130^ that enhanced levels of total carbohydrates and reducing sugar could be due to meddling of Pb ions with transportation through endodermis into plant cells causing severe toxicity. Pre-treatment with 24-EBL and SA led to further increase in content of sugars. Similar to amino acids, sugars also have stress mitigating properties *via* enhanced sequestration of ROS molecules as well as activation of antioxidant defense system^131^. It has been reported that sugars acts as osmolytes and provide protection to cells from metal toxicity^132,133^. These sugars not only help as osmolytes, participate in stabilization of cellular membranes and maintenance of turgor but also act as signaling molecules^134^.

## Conclusion

The present study indicates that co-application of 24-EBL and SA to *B*. *juncea* L. plants may enhance their potential to overcome Pb stress. The positive interpay between 24-EBL and SA led to elevation in total free amino acids, content of various amino acids (glutathione, proline, methionine etc.), which act as antioxidants and metal chelating compounds. These metabolites scavenge ROS and also result in reduced lipid peroxidation, nuclear and membrane damage. From the results, it is further concluded that 24-EBL and SA combined treatment has a significant role in regulating the balance of total carbohydrates, reducing sugars and osmoprotectants like trehalose, glycine betaine and proline which are involved in osmoregulation of plant cells. The co-application of 24-EBL and SA reduced Pb accumulation in plants that consequently results in lowering oxidative stress. Therefore, combined treatment of 24-EBL and SA can counter adverse effects of Pb toxicity through regulating various physiological processes. Further insight into mechanisms of 24-EBL and SA crosstalk pertaining to metal stress amelioration might provide better understanding of stress tolerance strategies in plants.
